# Examination of social determinants of health among patients with limited English proficiency

**DOI:** 10.1186/s13104-021-05720-7

**Published:** 2021-08-05

**Authors:** Austin Fischer, Joseph Conigliaro, Shaun Allicock, Eun Ji Kim

**Affiliations:** 1 Department of Medicine, Donald and Barbara Zucker School of Medicine at Hofstra/Northwell, 500 Hofstra Blvd, Hempstead, NY 11549 USA; 2 Institute of Health Innovations and Outcomes Research, Feinstein Institutes for Medical Research, 600 Community Drive Suite 403, Manhasset, NY 11030 USA; 3 Northwell Health, 5 Dakota Dr, New Hyde Park, NY 11042 USA

**Keywords:** Social determinants of health, Limited English proficiency, Health disparities, Population health

## Abstract

**Objectives:**

The purpose of this study is to examine the prevalence of social needs by English proficiency using data from Northwell Health’s social determinants of health screening program. The screening program evaluates 12 domains of social needs: material need, employment, medical-legal assistance, health insurance, public benefits, health literacy, transportation, medical care, utilities, housing quality, food security, and housing insecurity. We have identified patients to have limited English proficiency if they have selected a language other than English as their primary language.

**Results:**

The study population includes 92,958 individuals; of these, 83,445 (89.8%) patients are English proficient, and 9513 (10.2%) patients have limited English proficiency. A higher percentage of patients with limited English proficiency has social needs, including material need, employment, medical-legal assistance, health insurance, public benefit, health literacy, medical care, utility bill, poor housing quality, and food insecurity (all p-values < 0.05). In multivariable logistic regression models adjusting for sociodemographic information, LEP status (odds ratio = 1.36 [1.25–1.49]) has been associated with having social needs. These findings suggest that system-wide SDH screening and referral programs should identify ways to ensure capturing social needs among patients with limited English proficiency.

**Supplementary Information:**

The online version contains supplementary material available at 10.1186/s13104-021-05720-7.

## Introduction

Increasingly, the implications of social determinants of health (SDH) stretch beyond the individual, with the excess medical cost of inequalities estimated at $93 billion annually [1, 2]. Recent efforts have focused on systematically documenting SDH, particularly unmet social needs, as these factors have been shown to play a larger role in the health of an individual than either insurance status or access to care [3]. While the ultimate goal is to address systemic inequalities, evaluating population-level data offers the capacity for tailored intervention for vulnerable patients [4].

Limited English proficiency (LEP) has been established as a risk factor for poorer access to care, decreased healthcare utilization, and adverse health outcomes [5]. There is a need to understand the conditions impacting LEP patients, as this cohort has grown rapidly and now represents more than 25 million Americans [6]. This is particularly true in communities that have limited expertise, capital, and infrastructure to promote equity for this group. Despite past work investigating health outcome disparities in LEP patients, less is known about the presence of social needs in patients with LEP.

To fill this gap, using data from an integrated health network, we examined the presence of social needs by LEP status. In particular, we sought to evaluate the presence of social needs among patients with Spanish as a primary language. As the LEP population grows and expands to areas ill-equipped to support this group, understanding the prevalence of these individual needs will be key to addressing the social factors that ultimately impact their health.

## Main text

### Methods

#### Study population

The SDH screening program has been implemented in an inpatient setting at 11 acute-care hospitals throughout the Northwell Health system. The goal of the screening program is to estimate SDH risk by detecting both individual and community-level SDH prevalence. These hospitals are located throughout the New York City area and encompass a diverse population, including racial/ethnic minorities, immigrants, and traditionally underserved populations.

Our data consisted of SDH screenings conducted from June 25th, 2019 through February 29th, 2020. The SDH screening tool is embedded within nursing notes; upon admission, nurses complete the SDH screening along with patients’ demographic, clinical, and insurance information.

We included all adults (aged 18 years or older) that responded to the screening. For patients with multiple hospitalizations, we included their first hospitalization to prevent over-representation. We excluded patients with missing demographic information or preferred primary language.

#### Outcome measures

Our primary outcome measure was the presence of social needs. The SDH screening tool evaluates 12 areas of social needs, including material need, employment, medical-legal assistance, health insurance, public benefits, health literacy, transportation, medical care, utilities, housing quality, food security, and housing insecurity (Additional file 1: Table S1). Each response is documented as “yes” or “no”.

#### Independent variables

Our primary independent variable was LEP: patients who selected a language other than English as their primary language were categorized as LEP and those who preferred English as their primary language were categorized as non-LEP. Furthermore, patients with LEP were subcategorized into two cohorts: Spanish primary language and non-Spanish primary language. We also identified key demographic variables associated with the presence of social needs, including age, gender, race/ethnicity, and insurance. Responses for race/ethnicity were organized into White non-Hispanic, Black non-Hispanic, Hispanic, Asian, Native American/Alaskan, other/multiracial, and unknown/declined. Furthermore, health insurance responses were categorized into commercial, Medicare, Medicaid, self-pay, and unknown.

#### Statistical analyses

We performed a descriptive analysis of all participants, and then by English proficiency status. We calculated the mean and standard deviation for continuous variables and the percentage for categorical variables. Finally, to examine differences by English proficiency status, we calculated t-tests for continuous variables and chi-squared tests for categorical variables. We first compared English proficiency versus LEP, and then we further examined Spanish as a primary language because Spanish is the most common non-English language spoken in the United States. Lastly, we ran multivariable logistic regression models to examine patient characteristics associated with having at least one social need, including LEP status. Statistical analyses were conducted using SAS software (SAS Institute Inc., Cary NC, USA).

### Results

#### Population characteristic

**Fig. 1:**
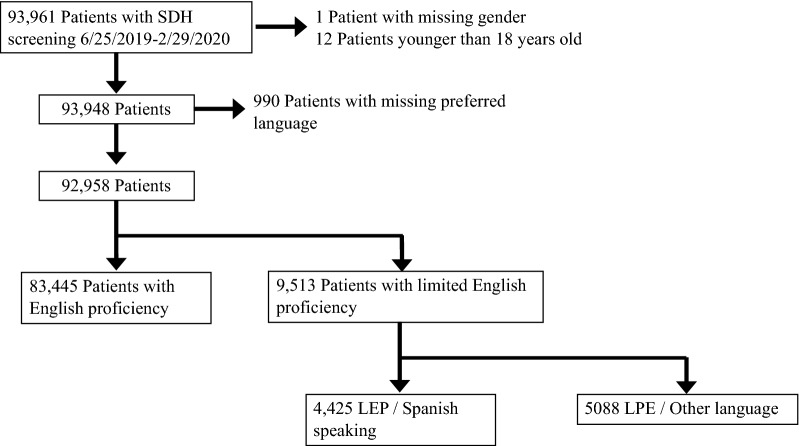
Flowchart of study population

The study population included 92,958 individuals who had responses to SDH screenings; of these, 83,445 (89.8%) patients were English proficient and 9513 (10.2%) patients had LEP (Fig. 1). There were differences in sociodemographic characteristics, including age, gender, race/ethnicity, and health insurance, between patients who are English proficient versus LEP (Table 1). A higher percentage of patients with LEP were Hispanic (40.8% versus 8.7%, p-value < 0.001) and had Medicaid (26.8% versus 10.4%, p-value < 0.001) compared to patients with English proficiency.

**Table 1:** Patient characteristics by English proficiency, n (%) for categorical variables and mean (standard deviation) for continuous variables

	All (n = 92,958)	English proficient (n = 83,445)	Limited English proficiency	
			All (n = 9513)^a^	Spanish (n = 4425)^b^
Age, years	65.2 (18.3)	64.8 (18.3)	69.0 (17.2)	63.8 (18.9)
Gender				
Female	49,211 (52.9%)	43,767 (52.5%)	5444 (57.2%)	2544 (57.5%)
Male	43,747 (47.1%)	39,678 (47.6%)	4069 (42.8%)	1881 (42.5%)
Race/ethnicity				
White, non-Hispanic	50,692 (54.5%)	48,917 (58.6%)	1775 (18.7%)	122 (2.8%)
Black, non-Hispanic	15,130 (16.3%)	14,753 (17.7%)	377 (4.0%)	14 (0.3%)
Hispanic	11,156 (12.0%)	7274 (8.7%)	3882 (40.8%)	3838 (86.7%)
Asian	6327 (6.8%)	4428 (5.3%)	1899 (20.0%)	6 (0.1%)
Native American/Alaskan	477 (0.5%)	368 (0,.4%)	109 (1.2%)	2 (0.05%)
Other/multiracial	6494 (7.0%)	5392 (6.5%)	1102 (11.6%)	355 (8.0%)
Unknown/decline	2682 (2.9%)	2313 (2.8%)	369 (3.9%)	88 (2.0%)
Health insurance				
Commercial	29,065 (31.3%)	27,533 (33.0%)	1532 (16.1%)	931 (21.0%)
Medicare	36,176 (38.9%)	32,194 (38.6%)	3982 (41.9%)	1649 (37.3%)
Medicaid	11,215 (12.1%)	8665 (10.4%)	2550 (26.8%)	1334 (30.2%)
Self-pay	735 (0.8%)	588 (0.7%)	147 (1.6%)	112 (2.5%)
Unknown	15,767 (17.0%)	14,465 (17.3%)	1302 (13.7%)	399 (9.0%)
Patient-reported social needs				
Material need	459/92,842 (0.5%)	391/83,348 (0.5%)	68/9494 (0.7%)	49/4417 (1.1%)
Employment	623/92,781 (0.7%)	528/83,291 (0.6%)	95/9490 (1.0%)	88/4414 (2.0%)
Medical legal assistance	390/92,764 (0.4%)	325/83,281 (0.4%)	65/9483 (0.7%)	53/4414 (1.2%)
Health insurance	1587/91,188 (1.7%)	1262/83,285 (1.5%)	325/9490 (3.4%)	246/4417 (5.6%)
Public benefit	1630/92,806 (1.8%)	1391/83,312 (1.7%)	239/9494 (2.5%)	169/4416 (3.8%)
Health literacy	1122/92,769 (1.2%)	765/83,284 (0.9%)	357/9485 (3.8%)	220/4415 (5.0%)
Public transportation	1382/92,782 (1.5%)	1225/83,291 (1.5%)	157/9491 (1.7%)	110/4413 (2.5%)
Medical care	788/92,784 (0.9%)	687/83,292 (0.8%)	101/9492 (1.1%)	80/4415 (1.8%)
Utility bill	1939/92,797 (2.1%)	1709/83,304 (2.1%)	230/9493 (2.4%)	182/4416 (4.1%)
Poor housing quality	628/92,872 (0.7%)	588/83,370 (0.7%)	40/9502 (0.4%)	30/4420 (0.7%)
Food insecurity	1018/92,850 (1.1%)	879/83,354 (1.1%)	139/9496 (1.5%)	114/4417 (2.6%)
Housing insecurity	1205/92,902 (1.3%)	1080/83,395 (1.3%)	125/9507 (1.3%)	100/4423 (2.3%)

^a^Chi-square test of similarity comparing patients with English proficiency versus patients with limited English proficiency were all less than 0.001, except public transportation and housing insecurity

^b^Chi-square test of similarity comparing Spanish speaking patients versus non-Spanish speaking patients were all less than 0.001

#### Social needs

Presence of social needs were: 0.5% material need, 0.7% employment, 0.4% medical-legal assistance, 1.7% health insurance, 1.8% public benefits, 1.2% health literacy, 1.5% transportation, 0.9% medical care, 2.1% utilities, 0.7% housing quality, 1.1% food security, and 1.3% housing insecurity. There also were differences in the presence of social needs based on LEP status (Table 1). A higher percentage of patients with LEP had social needs, including material need, employment, medical-legal assistance, health insurance, public benefit, health literacy, medical care, utility bill, poor housing quality, and food insecurity (all p-values < 0.05). Then we examined the presence of social needs among English proficient, Spanish primary language, and non-Spanish primary language; higher percentages of patients with Spanish as a primary language had social needs compared to patients with English proficiency (all p-values < 0.001). For example, 1.1% of patients with English proficiency versus 1.5% of patients with LEP and 2.6% of patients with Spanish as a primary language had food insecurity (p-value < 0.001).

#### Multivariable logistic regressions

We then examined patient characteristics associated with having at least one social need (Table 2). In the base model, racial minorities, except Asians (odds ratio (OR) = 0.86 [95% confidence interval = 0.76–0.98]), had increased odds of having social need(s) compared to non-Hispanic Whites. Being male (OR = 1.23 [1.17–1.30]) and having Medicare or Medicaid (OR = 2.17 [2.03–2.32]) were also associated with having social needs. When we included LEP status into the base model, LEP was associated with increased odds (OR = 1.36 [1.25–1.49]) of having social needs. When we further examined LEP status by Spanish speaking or not, Spanish as a primary language was associated with increased odds (OR = 1.74 [1.55–1.95]), and non-Spanish was not associated (OR = 1.02 [0.89–1.17]) with having social needs.

**Table 2:** Patient characteristics associated with having at least one social need (odds ratio [95% confidence interval])

	Base model	Base model + LEP	Base model + LEP (Spanish)
Race/ethnicity (reference: non-Hispanic White)			
Black	1.81 [1.68–1.94]	1.81 [1.68–1.94]	1.81 [1.68–1.94]
Hispanic	1.83 [1.70–1.98]	1.64 [1.51–1.79]	1.47 [1.34–1.62]
Asian	0.86 [0.76–0.98]	0.79 [0.70–0.90]	0.86 [0.76–0.98]
Other	1.38 [1.24–1.52]	1.31 [1.18–1.45]	1.32 [1.19–1.46]
Unknown/decline	1.82 [1.59–2.08]	1.75 [1.52–2.00]	1.77 [1.54–2.03]
Age	0.98 [0.98–0.99]	0.98 [0.98–0.98]	0.98 [0.98–0.98]
Male	1.23 [1.17–1.30]	1.24 [1.18–1.31]	1.24 [1.18–1.31]
Health insurance (reference: commercial health insurance)			
Medicare/Medicaid	2.17 [2.03–2.32]	2.14 [2.00–2.29]	2.14 [2.00–2.29]
No health insurance/unknown	1.05 [0.96–1.16]	1.05 [0.95–1.15]	1.06 [0.65–1.16]
LEP (reference: English speaking)			
LEP		1.36 [1.25–1.49]	
Spanish speaking			1.74 [1.55–1.95]
Non-Spanish LEP			1.02 [0.89–1.17]

### Discussion

Using data from a large integrated health system, this study illustrated that LEP status was associated with an increased presence of social needs, including material need, employment, medical-legal assistance, health insurance, public benefit, health literacy, medical care, utility bill, poor housing quality, and food insecurity. Additionally, patients with Spanish as a primary language had increased social needs across all domains, including public transportation and housing insecurity. This finding persisted even after adjusting for covariates in multivariable logistic regression analysis. As SDH screening is expanding at hospitals and health systems, it will be vital to capture social needs among LEP patients. Ultimately, these data can be utilized to understand the root causes of these health disparities—the identification of social needs associated with healthcare utilization and health outcomes [7]. Furthermore, hospitals and health systems can allot existing limited resources to improve patients’ health outcomes.

Predictably, the root of LEP disparities centers on language. Language barriers are known to perpetuate cultural barriers, as limited understanding of American culture can prevent the assimilation of immigrants and refugees. In turn, the combination of lingual and cultural barriers restricts LEP individuals from accessing and utilizing medical care and connecting to community resources [5, 8]. Despite the recognition of these phenomena, the responsibility of resolving disparities in LEP has by and large fell onto the patient. While the identification of subgroups most vulnerable to adverse outcomes is key to guiding focused intervention, what is needed now is a thorough understanding of the interventions to address social needs among patients with LEP.

In clinical settings, interventions to address social needs have centered on recruiting assistance from non-profits, expanding culturally competent practices, and increasing patient engagement [9–12]. Efforts from the voluntary sectors have been particularly useful, as these organizations are positioned to be the bridge between the clinic and resources necessary to address social needs. For example, the NOWPOW program links together a patient’s electronic health record with community-based databases to generate prescriptions for patients in need [10, 13]. Also, there is great utility in expanding culturally competent practices, such as the use of interpreters and providing language-appropriate materials. Despite an extensive amount of literature demonstrating the efficacy of trained interpreters in improving health outcomes, reducing healthcare costs, and increasing health literacy, patients with LEP are frequently communicated without an interpreter [14]. Furthermore, patient engagement has been cited as a method for addressing healthcare disparities, but has failed to incorporate populations likely to experience disparities onto patient/family advisory groups. Therefore, the path to addressing social needs within the LEP population will be an interdisciplinary approach, paved through combined local and federal intervention.

### Conclusion

Both LEP status and Spanish as a primary language were associated with an increased presence of social needs. While legislation exists to ensure care equality, gaps in implementation place the responsibility on providers, hospital systems, and communities to be forerunners in screening and addressing social needs. Accurately capturing social needs among patients with LEP to ensure subsequent referral to appropriate services is paramount. Additionally, research is needed investigating the presence of underreporting in SDH screenings of LEP populations, as well as exploring the efficacy of interventions.

## Limitation

There are several study limitations. Social needs might have been underreported, especially among LEP patients with societal and legal concerns. Individual social needs are being documented as yes/no responses; “no” to each social need question can be due to a lack of social need or a missing response. Therefore, the actual prevalence of social needs may be higher than documented in the electronic health record. We also identified LEP based on their preferred language, which can result in identifying patients who are fluent in English and non-English language to be labeled as LEP patients.

## Supplementary Information

**Additional file 1: Table S1.** Social determinants of health screening questions.

## Data Availability

The data sets used and/or analyzed during the current study are available from the corresponding author upon request.

## Abbreviations

SDH: Social determinants of health
LEP: Limited English proficiency
