# Supplementary material for: Examination of social determinants of health among patients with limited English proficiency
Source: BMC Res Notes. 2021 Aug 5;14:299. doi: 10.1186/s13104-021-05720-7 (PMC8340469; doi:10.1186/s13104-021-05720-7)
Supplement: Supplementary file 1 — Additional file 1: Table S1. Social determinants of health screening questions. [file 13104_2021_5720_MOESM1_ESM.docx]

| Table S1. Social determinants of health screening questions | |
| --- | --- |
| Domain | Question |
| Living Environment | Do you worry that the place you are living is making you sick? |
| Housing | Do you worry that in the next two months you/your family may not have a safe place to live? |
| Finance | Do you worry that you will run out of food before you get money to buy more? |
| Utility | Do you have trouble paying your bills? |
| Child Care | Do you/family member need any of the following: clothing, diaper, car seats, school supplies, other? |
| Public Benefit | Do you need help getting public benefits such as food stamps, WIC, welfare, disability income, other? |
| Medical Care | Do you ever skip medications that you need, or going to the doctor, to save money? |
| Employment | Do you need help finding a job? |
| Health Literacy | Do you ever need help understand what your doctor tells you, or help reading health information? |
| Legal | Do you need help from a lawyer with the following: housing, immigration, custody, child support, other? |
| Health Insurance | Do you need help getting dental or health insurance? |
| Transportation | Do problems with transportation stop you from getting to doctor visits or getting our medication? |
